# Feasible attack on detector-device-independent quantum key distribution

**DOI:** 10.1038/s41598-017-00531-y

**Published:** 2017-03-27

**Authors:** Kejin Wei, Hongwei Liu, Haiqiang Ma, Xiuqing Yang, Yong Zhang, Yongmei Sun, Jinghua Xiao, Yuefeng Ji

**Affiliations:** 1 School of Science and State Key Laboratory of Information Photonics and Optical Communications, Beijing University of Posts and Telecommunications, Beijing, 100876 China; 2 Guangxi Key Laboratory for Relativistic Astrophysics, School of Physics Science and Technology, Guangxi University, Nanning, 530004 China; 3 School of Science, Beijing Jiaotong University, Beijing, 100044 China

## Abstract

Recently, to bridge the gap between security of Measurement-device-independent quantum key distribution (MDI-QKD) and a high key rate, a novel protocol, the so-called detector-device-independent QKD (DDI-QKD), has been independently proposed by several groups and has attracted great interest. A higher key rate is obtained, since a single photon bell state measurement (BSM) setup is applied to DDI-QKD. Subsequently, Qi has proposed two attacks for this protocol. However, the first attack, in which Bob’s BSM setup is assumed to be completely a “black box”, is easily prevented by using some additional monitoring devices or by specifically characterizing the BSM. The second attack, which combines the blinding attack and the detector wavelength-dependent efficiency, is not explicitly discussed, and its feasibility is not experimentally confirmed. Here, we show that the second attack is not technically viable because of an intrinsically wavelength-dependent property of a realistic beam splitter, which is an essential component in DDI-QKD. Moreover, we propose a feasible attack that combines a well-known attack—detector blinding attack with intrinsic imperfections of single-photon detectors. The experimental measurement and proof-of-principle test results confirm that our attack can allow Eve to get a copy of quantum keys without being detected and that it is feasible with current technology.

## Introduction

Quantum key distribution (QKD) enables two legitimate parties, usually called Alice and Bob, to share a private key through a quantum channel, which is possible in the presence of an eavesdropper, Eve^1, 2^. Ideal QKD protocols, guaranteed by the laws of quantum mechanics, have been proved to be unconditionally secure without imposing any restrictions on the computational power of Eve^3, 4^. However, real-life implementations of QKD inevitably contain certain imperfections leaving some vulnerable loopholes (the so-called side channels). Indeed, such loopholes have been exploited by Eve and have led to various subtle attacks for real-life QKD systems^5–16^. For example, in the security proof of QKD, a key assumption is that two legitimate parties encode their signals without errors, which is violated in a real QKD system. Xu *et al*. have experimentally shown that Eve can exploit such loopholes to launch a phase-remapping attack to steal secure keys without being detected^10^. See ref. 17 for a recent review.

To build loophole-free QKD systems, the one crucial solution is to develop device-independent QKD protocols^18–20^, which can remove all loopholes due to imperfect devices. Among them, the measurement-device-independent (MDI) QKD protocol, which is more practical in comparison to other device-independent protocols, has received great attention. MDI-QKD can automatically remove all side channels from measurement apparatus, which are undoubtedly the most critical part of QKD systems. Several groups have experimentally demonstrated the practicality of MDI-QKD with current technology^21–27^. Ref. 28 is a recent review on this topic. However, setups of MDI-QKD, which require a two-photon interference from two individual lasers, are rather complicated and lead to a lower key rate comparing to conventional QKD, such as decoy-state BB84 QKD^29, 30^.

Recently, to bridge the gap between security of MDI-QKD and a high key rate, a novel protocol, the so-called detector-device-independent QKD (DDI-QKD)^31^, has been independently proposed by several groups and has attracted great interest^32–34^. Instead of exploiting two-photon interference in MDI-QKD, this protocol utilizes an untrusted single-photon Bell state measurement (BSM) setup^35^. Consequently, as declared in DDI-QKD, it can still be immune to all detector side-channel attacks, which is the major advantage of MDI-QKD, with a comparable performance of conventional QKD. Subsequently, Qi reported two attacks for this protocol and showed that additional assumptions on the BSM setup are required to reach the claimed DDI-QKD security level^36^. To launch the first attack, the BSM located inside Bob’s laboratory is assumed to be completely a “black box” (as the security assumption claimed in refs 32–34), which led to leak of “unwanted” information to Eve. Nevertheless, this attack is easily removed by either introducing additional apparatus to monitor Bob’s input signals^32^ or specifically characterizing the BSM^34^. The second attack (we call it the *wavelength*-*dependent attack* since it exploits the detector wavelength-dependent efficiency), combining a well-known detector blinding attack and the detector wavelength-dependent efficiency, allows Eve to learn the total key string without introducing any errors. In the attack, Eve employs bright light to blind Bob’s detectors and then controls which detector clicks by carefully tailoring the wavelength of a superimposed bright pulse into the bright light. If detectors in the BSM have different wavelength-dependent efficiencies, Eve exploits such an imperfection to cover unusual double-click rate s caused by her attack. However, ref. 36 does not explicitly discuss the second attack. For example, how can Eve blind four detectors? More importantly, it is unknown whether the attack employing the detector wavelength-dependent efficiency is experimentally feasible. We note that, more recently, a simple implementation of DDI-QKD has been experimentally reported^37^.

Here, we show that the wavelength-dependent attack in ref. 36 is not technically viable because of the harm caused by an intrinsically wavelength-dependent property of a realistic beam splitter, which is an essential component in DDI-QKD. Moreover, we propose a feasible attack, the same as the wavelength-dependent attack that is based on the well-known detector-blinding attack. We explain how Eve blind four detectors in detail. To avoid an unusual double-click rate, different from the wavelength-dependent attack, we utilize another imperfection that two practically blinded detectors respond differently to the same blinding power. Our experimental measurement and proof-of-principle test results confirm that our attack can allow Eve to get a copy of a quantum key without being detected and that it is feasible with current technology. We remark that our attack is not against the wavelength-dependent attack; strictly speaking, we propose an alternative solution to the problem of double clicks in the wavelength-dependent attack and experimentally confirm its feasibility.

## Results

### DDI-QKD

**Figure 1:**
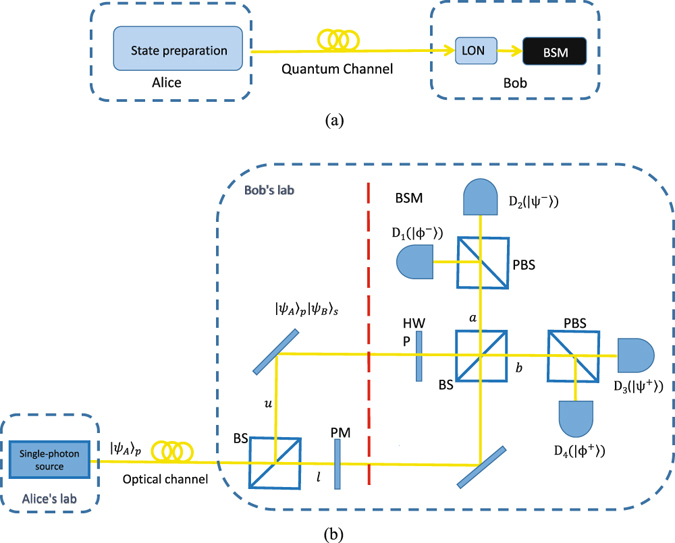
(**a**) Schematic diagram of DDI-QKD. LON is the linear optical network, and BSM is the Bell state measurement. (**b**) Schematic diagram of a specified example of DDI-QKD proposed in ref. 33. BS: 50:50 beam splitter, BSM: Bell state measurement, HWP: half-wave plate, PM: phase modulator, PBS: polarizing beam splitter, and *D*_*i*_, with $i\in \{1,2,3,4\}$: single-photon detectors in the BSM setup. In Bob’s laboratory, the areas before and after the red dashed line denote the LON and the BSM setup, respectively. For the ideal case, a click on each detector *D*_*i*_ corresponds to the projection on one of the four Bell states. |*ψ*_*A*_〉_*p*_ is one of the four BB84 polarization states prepared by Alice, and |*ψ*_*B*_〉_*s*_ are Bob’s encoding bit in spatial mode. Symbols *u*, *l* and *a*, *b* denote two paths of the first and second BS, respectively. In practice, Alice prepares |*ψ*_*A*_〉_*p*_ by generating phase-randomized weak coherent pulses which can be modelled as Poissonian (with parameter *u*) mixture of photon number states in different BB84 polarization states^1, 2, 29^.

In DDI-QKD (as shown in Fig. 1(a)), Alice generates a single photon in one of the four BB84 polarization states |*ψ*_*A*_〉_*p*_ and then sends it to Bob over an insecure quantum channel. Once Bob receives the photon, he exploits a trusted linear optical network (LON), which includes some linear optical components for manipulating the state of an incoming photon, to encode his random bit |*ψ*_*B*_〉_*s*_ in a different degree (say spatial) of freedom of the photon. Afterwards, the photon is detected by an untrusted single-photon BSM setup, which is considered to be a “black box” (this means that the BSM, for the worst case, is controlled by Eve), and the measurement result is reported to Bob. Finally, through an authenticated classical channel, Bob broadcasts, which photons have a successful BSM click together with the corresponding Bell state gained and the basis information. Then, Alice and Bob estimate the quantum bit error rate (QBER) for the data where they choose the same basis. If the QBER is below the secure threshold, they perform error correction and privacy amplification to distill the secure key. To clearly describe our attack, we use the proposed protocol in ref. 33 as an example. The implementation of the protocol is shown in Fig. 1(b). To encode the information in the spatial mode, Bob sends the receiving photon to a 50/50 beam slitter (BS) and applies a phase *φ*_*B*_ on the lower arm (represented by *l*) by a phase modulator (PM). A single-photon BSM setup contains a half-wave plate (HWP) on the upper arm (represented by *u*), followed by a 50/50 BS to combine the two arms, and two polarizing beam splitters (PBSs) followed by four single-photon detectors (SPDs). With this structure shown in Fig. 1(b), a click on each SPD denotes the projection on one of the Bell states. In practice, Alice prepares |*ψ*_*A*_〉_*p*_ by generating phase-randomized weak coherent pulses which can be modelled as Poissonian (with parameter *μ*) mixture of photon number states in different BB84 polarization states^1, 2, 29^. The density matrix of the state can be written as ${\rho }_{A}={\sum }_{i=0}^{\infty }\frac{{\mu }^{i}}{i!}{e}^{-\mu }|i\rangle \langle i|$, where |*i*〉 〈*i*| is the density matrix of the *i*–photon state for *i* = 0, 1, …. When *μ* is small, the contribution of the *n* = 1 state to a mixture of the photon number states dominates that of the *n* > 2 multiphoton states. Although the possible implementations of the DDI protocol in refs 32–34 are somewhat different, our attack is feasible on all of them since they all employ four SPDs to perform the BSM.

### Wavelength-dependent attack

**Figure 2:**
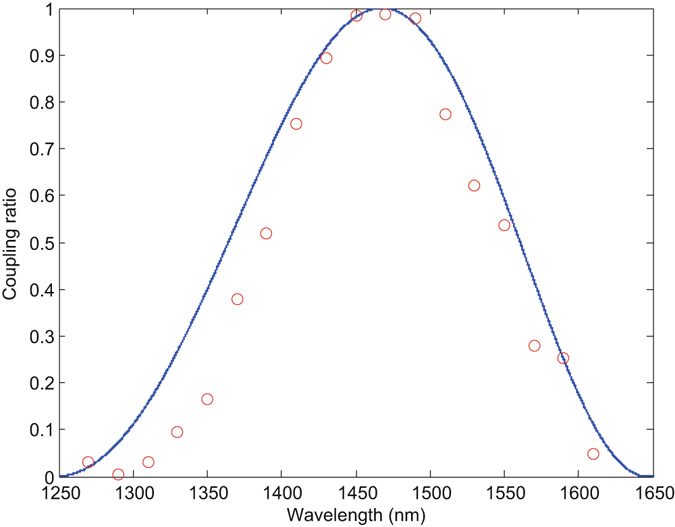
Relationship between the wavelength of the source and the coupling ratio. The coupling ratio of the BS is defined as $r=\frac{{I}_{port1}}{{I}_{port1}+{I}_{port2}}$, where *I*_*port*1_ (*I*_*port*2_) is the output intensity of BS output 1(2). The theoretical relationships are based on the coupling model *r* = *F* ^2^ sin^2^(*Cω*/*F*) given in ref. 11, where *F* ^2^ is the maximal coupled power, *C* is the coupling coefficient, and *ω* is the heat source width. The red dot is the experimental measurement result, and the curve is the theoretical result. The coupling ratio changes from 0.003 to 0.968 with the wavelength changing from 1290 nm and 1470 nm. (The figure is reproduced with permission from ref. 11).

To highlight the feasibility of our proposed attack, first, we show that the wavelength attack is failed because of its applied strategy (the detector wavelength-dependent efficiency) to remove an unusual double-click rate. As discussed in the wavelength-dependent attack^36^ or a similar analysis in the following subsection 2, Eve could use the imperfection-detector wavelength-dependent efficiency in the BSM to deal with an unusual double-click rate resulted by detector controlling, i. e., Eve could reduce the double-click rate by carefully tailoring the wavelength of the superimposed light. This solution will work well if all fiber-optic components in Bob’s laboratory are wavelength-independent. Unfortunately, in practice, a part of the fiber-optic components, such as BSs and PMs, are wavelength-dependent and affect which detector clicks with the varying wavelength of the superimposed light. For instance, a realistic BS is generally manufactured using fused biconical taper technology, and the coupling ratio is commonly wavelength-dependent. Figure 2 shows the relationship between the wavelength of a source and the coupling ratio of a fused biconical taper 50/50 BS. The coupling ratio can be easily checked to vary nonlinearly with the wavelength, and the optimal coupling ratio is reached at the central wavelength of 1550 nm. For simplicity, in the wavelength-dependent attack, to reduce the unusual double-click rate, the imperfection should satisfy that the detector *D*_2_(*D*_3_) clicks at the central wavelength and detector *D*_1_(*D*_4_) clicks at a different wavelengths *λ* with an unbalanced coupling ratio *r*. Here, we define *r* = *P*_*i*_/(*P*_*i*_ + *P*_*j*_), where $i\in \{u,a\}$ and $j\in \{l,b\}$ represent the paths of BS in Fig. 1b. Suppose that the superimposed light that Eve resends to Bob are in the form |2*α*_*e*_〉, according to DDI-QKD (a similar and detailed procedure is shown in following subsection 2), the intensities arriving at different detectors in the wavelength-dependent attack can be expressed as

1 $$\begin{array}{lll}-{|[r+\mathrm{(1}-r){e}^{i({\phi }_{e}+{\phi }_{B})}]{\alpha }_{e}\rangle }_{{D}_{1}} & \otimes  & -{|[r{e}^{i{\phi }_{e}}+\mathrm{(1}-r){e}^{i{\phi }_{B}}]{\alpha }_{e}\rangle }_{{D}_{2}}\\  & \otimes  & i{|\sqrt{r\mathrm{(1}-r)}({e}^{i{\phi }_{e}}-{e}^{i{\phi }_{B}}){\alpha }_{e}\rangle }_{{D}_{3}}\\  & \otimes  & i{|\sqrt{r\mathrm{(1}-r)}(1-{e}^{i({\phi }_{e}+{\phi }_{B})}){\alpha }_{e}\rangle }_{{D}_{4}},\end{array}$$

where *φ*_*e*_ and *φ*_*B*_ represent the phase choices of Eve and Bob, respectively, and *D*_*i*_, with $i\in \{1,2,3,4\}$, denote Bob’s detectors. From Eq. (1), we know that the intensities arriving at each detector are extremely dependent on the coupling ratio *r*. With random phase modulations, the counts of the four detectors are distributed evenly only if the coupling ratio *r* equals 0.5. For a sharp comparison, we suppose that *r* = 1 corresponds to the wavelength *λ* = 1260 *nm* in Fig. 2. Eq. (1) converts into

2 $$-{|{\alpha }_{e}\rangle }_{{D}_{1}}\otimes -{|{e}^{i{\phi }_{e}}{\alpha }_{e}\rangle }_{{D}_{2}}.$$

The light only arrives at the two detectors at the output *a* regardless of the basis choice of Bob and Alice. Consequently, Bob can easily detect the wavelength-dependent attack by checking whether the four detectors respond equally to the four Bell states.

### Proposed attack

Our proposed attack can be summarized with the following three steps:

#### Step 1 - Detector blinding

In DDI-QKD, Alice prepares randomly quantum signals in the *X* or *Y* basis at a single-photon level. However, in our attack, Eve employs bright light |2*β*_*e*_〉_*α*_ in the *Z* basis to force Bob’s detectors into the so-called linear mode^8^, where the subscript *α* denotes an eigenstate in the *Z* basis. In detail, Eve intercepts Alice’s signals and then sends bright light |2*β*_*e*_〉_*H*_ (for simplicity, we assume the polarization of Eve’s light horizontal) to Bob. By using the LON, Bob can yield the BB84 states ${|{\psi }_{B}\rangle }_{s}=(i|u\rangle +{e}^{i{\phi }_{B}}|l\rangle )/\sqrt{2}$ in spatial modes, where ${\phi }_{B}\in \{0,\pi \mathrm{/2},\pi ,3\pi \mathrm{/2}\}$. Therefore, after passing the LON, |2*β*_*e*_〉_*H*_ becomes ${|2{\beta }_{e}\rangle }_{H}\otimes {|{\psi }_{B}\rangle }_{s}$. When the light enters the BSM setup, it first is made unitary transformations |*Hu*〉 → |*Vu*〉 and |*Vu*〉 → |*Hu*〉 on the upper arm by the HWP. Hence, the state transforms into $i{|\sqrt{2}{\beta }_{e}u\rangle }_{V}\otimes {e}^{i{\phi }_{B}}{|\sqrt{2}{\beta }_{e}l\rangle }_{H}$. After transmitting through the second BS, according to the unitary transformation $|u\rangle \to (i|a\rangle +|b\rangle )/\sqrt{2}$ and $|l\rangle \to (|a\rangle +i|b\rangle )\sqrt{2}$, the state can be written as

3 $$i{|{\beta }_{e}\rangle }_{{D}_{1}}\otimes i{|{e}^{i{\phi }_{B}}{\beta }_{e}\rangle }_{{D}_{2}}\otimes {|{e}^{i{\phi }_{B}}{\beta }_{e}\rangle }_{{D}_{3}}\otimes {|{\beta }_{e}\rangle }_{{D}_{4}},$$

In Eq. (3), it does not matter which modulated phase is chosen by Bob as the intensities entering the four SPDs are always the same. Thus, by elaborately tailoring the light, Eve can successfully blind Bob’s detectors, forcing the four detectors to work in the so-called linear operation mode. In this mode, the SPDs do not respond to a receiving single photon, but remain sensitive to bright light with a classical optical power threshold *P*_*th*_. We remark that this is not the only way to blind the detectors; alternative schemes, such as thermal blinding, have been reported^38–40^.

#### Step 2 - Detector controlling

After the blinding process in step 1, Eve performs an intercept-resend attack on the signals transmitted from Alice. That is, she intercepts the transmitted signals and measures each of them in one randomly chosen basis from the BB84 bases, and then she prepares a new signal according to her measurement result and sends it to Bob. Bob will get a detection event only if his active basis choice coincides with that of Eve. Here, without any loss of generality, we suppose that Eve resends a superimposed light pulse |2*α*_*e*_〉_*R*_ to control Bob’s detectors, where the subscript *R* represents the BB84 states $(|H\rangle +{e}^{i{\phi }_{e}}|V\rangle )/\sqrt{2}$ re-prepared by Eve. With a similar analysis in step 1, after passing the LON and the second BS, the state becomes

4 $$\begin{array}{lll}\frac{1}{\sqrt{2}}i{|[1+{e}^{i({\phi }_{e}+{\phi }_{B})}]{\alpha }_{e}\rangle }_{{D}_{1}} & \otimes  & \frac{1}{\sqrt{2}}i{|({e}^{i{\phi }_{e}}+{e}^{i{\phi }_{B}}){\alpha }_{e}\rangle }_{{D}_{2}}\\  & \otimes  & \frac{1}{\sqrt{2}}{|({e}^{i{\phi }_{e}}-{e}^{i{\phi }_{B}}){\alpha }_{e}\rangle }_{{D}_{3}}\\  & \otimes  & \frac{1}{\sqrt{2}}{|(1-{e}^{i({\phi }_{e}+{\phi }_{B})}){\alpha }_{e}\rangle }_{{D}_{4}}\mathrm{.}\end{array}$$

According to the Eq. (4) above, we can obtain the observed intensity arriving at each of Bob’s four detectors. If Bob and Eve use the same basis, only two out of the four detectors have the same arriving intensity (one-half of the total intensity). When they have an incompatible basis choice, all the four detectors share the intensity evenly (a quarter of the total intensity). To highlight it, as an example, Table 1 lists the observed intensities arriving at each of Bob’s four detectors when Eve sends |+〉 and Bob measures it with different bases. Here, we suppose that the classical optical power threshold of Bob’s blinded detectors *P*_*th*_ meets ${|\alpha |}^{2}\mathrm{/2} < {P}_{th} < {|\alpha |}^{2}$. From this table, we easily find that when Bob chooses |±*i*〉, which is a different basis from Eve, his blinded SPDs cannot have any detection events; when Bob chooses the same basis as Eve, two of his four SPDs may have clicks. However, it will cause an unusual double-click rate that ensures Bob is vigilantly aware of the presence of Eve and easily prevents our attack with random bit assignments for double clicks^41^. Consequently, to successfully perform the attack, it is necessary to find a solution to ensure that only one detector has a click.

**Table 1:** Observed intensity arriving at each of Bob’s four detectors when Eve sends |+〉.

Eve	Bob	*D*_1_ (|*ψ* ^−^〉)	*D*_2_ (|*ϕ* ^−^〉)	*D*_3_ (|*ϕ* ^+^〉)	*D*_4_ (|*ψ* ^+^〉)
+	+	1	1	0	0
	−	0	0	1	1
	+*i*	0.5	0.5	0.5	0.5
	−*i*	0.5	0.5	0.5	0.5

Here, we suppose that the classical optical power threshold of Bob’s blinded detectors *P*_*th*_ meets |*α*|^2^/2 < *P*_*th*_ < |*α*|^2^. The intensities are normalized by |*α*|^2^.

#### Step 3 - Reducing the double-click rate

**Figure 3:**
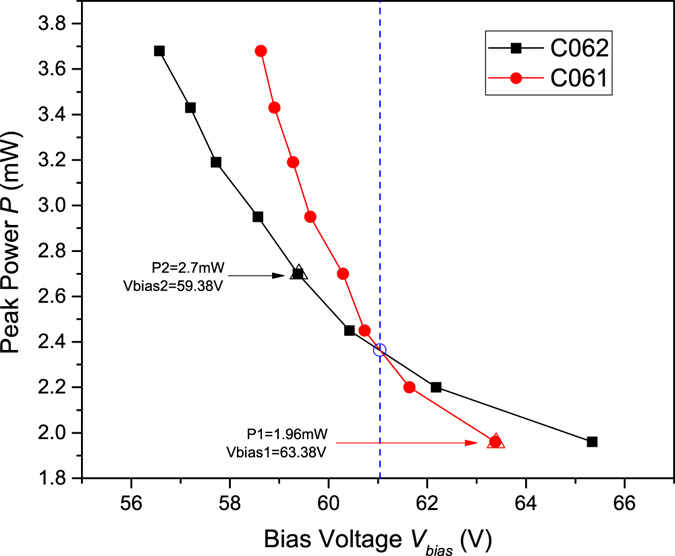
Click thresholds versus biased voltage of the APDs in two different SPDs C062 and C061. Here, for a particular biased voltage of APD Vbias, each point in the red (black) curves represents the minimum value of the peak power of SPD C061 (C062). The blue circle is the cross point of the two curves. The triangles are two different combinations in the feasibility test.

**Figure 4:**
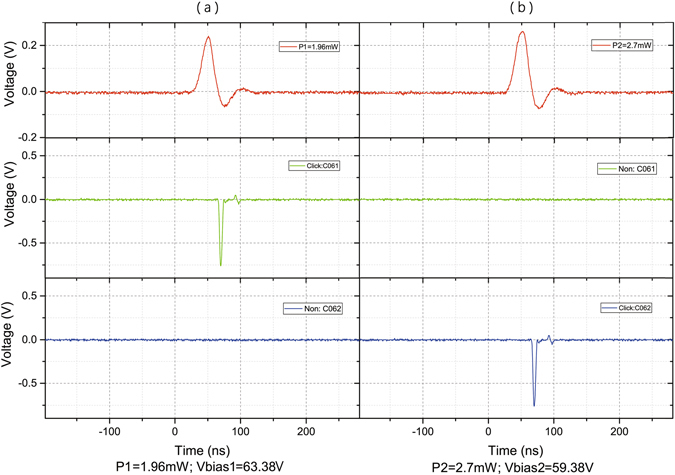
Electrical signal oscillograms when the APDs in SPDs Co62 and C061 are controlled by the trigger pulse peak power with a corresponding biased voltage. (**a**) The values *P* = 1.96 *mW* and *V* *bias* = 63.38 *V* cause a detection event in detector C061, however, never cause it in detector C062. (**b**) In contrast, the values *P* = 2.7 *mW* and *V* *bias* = 59.38 *V* cause a detection event in detector C062, however, never cause it in detector C061.

To reduce the usual double-click rate, in our attack, we apply an intrinsic imperfection of SPDs that real single-photon detectors respond differently to the same blinding power. As shown in Fig. 3 (see Methods section for the detailed measurement setup), for a particular biased voltage of avalanche photodiodes (APDs) Vbias in two different SPDs, the click thresholds are different. In the original blinding attack, the blinding is caused by the drop of the bias voltage such that the APD operates in the linear mode. Hence, in our measurement, we directly change the bias voltage of the APDs in the two SPDs and record corresponding click thresholds. Especially, with the increase in Vbias, the click threshold curves for the two detectors cross (see the dashed line in Fig. 3). Before the dashed line, the threshold of detector C062 is less than that of detector C061; however, after the dashed line, the threshold of detector C062 is larger than that of detector C061. Hence, Eve can simply choose a combination of P and Vbias before (after) the dashed line, such that a click occurs only on detector C062 (C061) and no click occurs on detector C061 (C062). By doing this, Eve can easily reduce the usual double-click rate. To demonstrate the feasibility of our attack, we control the two detectors with two different combinations of P and Vbias. The results are shown in Fig. 4. The values *P*1 = 1.96 *mW* and *V* *bias*1 = 63.38 *V* cause a detection event in detector C061, however, never cause it in detector C062. In contrast, the values *P*2 = 2.7 *mW* and *V* *bias*2 = 59.38 *V* cause a detection event in detector C062, however, never cause it in detector C061.

It seems that Eve can use another independent imperfection of detectors—detector efficiency mismatch—to reduce an unusual double-click rate. However, there is no concrete proof that the detector still has the efficiency mismatch when it works in the linear mode. It is worth noting that DDI-QKD is naturally secure against a time-shift attack^7^, which is based on the fact that two practical SPDs have detection-efficiency mismatch in time domain and each SPD denotes respectively one of bits 0 and 1. However, such criterion are not suitable in DDI-QKD, in which the bits each SPD denotes is varying with Alice’s and Bob’s chosen bases. A related scheme^42^, in which Alice and Bob will get conclusive bits only when they used the different basis for measurement, is strongly similar to DDIQKD and is also immune to the time-shift attack. However, our attack is still working for this scheme, that is, detector will keep silence when Bob’s active basis choice did not coincide with that of Eve.

### Schematics of hacking scheme

**Figure 5:**
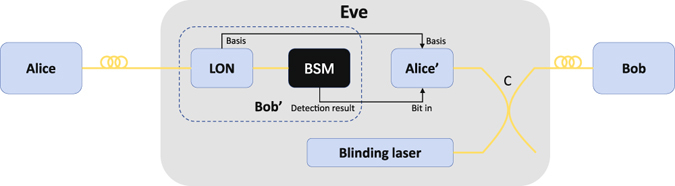
Schematics of hacking scheme. Eve consists of copies of Alice (Alice’) and Bob (Bob’), which share bit and basis settings and a blinding laser. C: optical coupler.

Figure 5 shows the proposed hacking scheme. Eve consists of copies of Alice (Alice’) and Bob (Bob’), which share bit and basis settings and a blinding laser. To perform our attack, Eve intercepts the state of Alice and randomly measures it in one of the two bases *X* and *Y* using the same devices as Bob. Eve then prepares new signals with detection results and the chosen basis from Bob’, overlaps them to a continuous-wave blinding laser which keeps Bob blind via an optical coupler C. Such detector blinding procedure and detector controlling have been successfully tested in QKD systems under laboratory or realistic condition^9, 43^. The critically implemented part of our attack is whether Eve can prepare the new signals to make only one of detectors in Bob have clicks. As we analyse in *step 3*, the answer is “yes”. For simplicity, suppose that Eve wants to force a click only on detector *D*_1_ and no click on detector *D*_2_ illustrated in Fig. 1(b). If the behaviour of the detector *D*_1_ (*D*_2_) coincides with the red (black) curves illustrated in Fig. 3, the values *P* = 1.96 *mW* and *V* *bias* = 63.38 *V* are an combination that meet the requirements. Similarly, if *P* = 2.7 *mW* and *V* *bias* = 59.38 *V*, Eve can cause a detection event in detector *D*_2_, however, never cause it in detector *D*_1_. The corresponding experimental tests for these examples are shown in Fig. 4. In our experiment, a full demonstration of the proposed hacking scheme has not been performed. The detector blinding procedure, which had been successfully tested in previous works, has not been repeated. The detector controlling, proofing that Eve can reduce usual double-click rates, has been performed. It worth mentioning that, although the results shown in Fig. 3 are gained by measuring a home-made detector circuit, we note that, in a recent work, Huang *et al*.^44^ show that SPDs in commercial QKD system Clavis2 have similar behaviours. Futhermore, our attack is valid for the scheme proposed in ref. 34. It is easy to check that the intensities arriving at Bob’s four detectors are the same if the initial polarization of the bright light of Eve is $|R\rangle =(|H\rangle +|V\rangle )/\sqrt{2}$. By careful tailoring the intensities of the bright light and superimposed light pulse according to steps 2 and 3, Eve can successfully hack the scheme without being detected.

## Discussion

In summary, we show that the wavelength-dependent attack in ref. 36 is not technically viable because of an intrinsically wavelength-dependent property of a realistic beam splitter, which is an essential component in DDI-QKD. Moreover, we propose a feasible attack, the same as the wavelength-dependent attack that is based on the well-known detector-blinding attack. We explain how Eve blinds four detectors in detail. To avoid an abnormal double-click rate, different from the wavelength-dependent attack, we utilize another imperfection in real single-photon detectors. An experimental measurement and a proof-of-principle test are performed, and their results confirm that our attack is feasible with current technology. To prevent our attack as well as the wavelength-dependent attack, one specific countermeasure is that Bob introduces additional filtering and monitoring devices with the same idea of QKD with an untrusted source for plug & play system^45^. Another countermeasure is the so-called random-detector efficiency, in which the efficiency of SPDs is randomly varied^46^. We remark that while DDI-QKD is not strictly secure as MDI-QKD, it is more secure than traditional QKD protocols, such as BB84, and is the best for certain practical applications.

## Methods

### Measurement method

**Figure 6:**
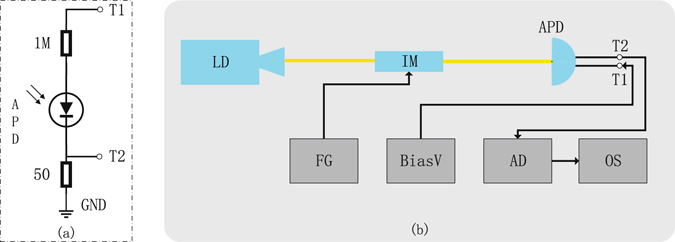
(**a**) Detector circuit diagram. Tap T1: analog tap of the APD bias voltage with a biased resistance of 1 MΩ. T2: analog tap of avalanche signals with a load resistance of 50 Ω. (**b**) LD: laser diode, IM: intensity modulator, FG: function generator, BiasV: home-made DC power, AD: amplifier discriminator, and OS: oscilloscope.

Figure 6(a) shows the detector circuit diagram in our experiment. The APD is biased by a high voltage from analog tap T1 with a biased resistance of 1 MΩ. Tap T2 is an analog tap of avalanche signals with a load resistance of 50 Ω. We do not re-engineer exact detector circuit in commercial SPDs, which contain filter circuits and a fast comparator. Figure 5(b) shows the measurement setup used to measure intrinsic imperfections of APDs. To produce trigger pulses, a laser diode sends light pulses, and then they are attenuated by an intensity modulator controlled by directly applying the pulsed voltage from a function generator (Tektronix AFG3022C). The detector blinding is simulated by directly applying a high voltage for tap T1 from a home-made DC power. An amplifier discriminator (ORTEC 9302) and an oscilloscope (Tektronix TDS 2024B) are used to monitor the avalanche signals. The two APDs are both from Princeton Lightwave. The breakdown voltage of the first APD (Serial number 1444C061), denoted as C061 in Fig. 3, is 72.41 V at 25 °C, and that of the second APD (Serial number 1444C062), denoted as C062, is 71.25 V at 25 °C. In the imperfection measurement, we record the click threshold in the biased voltage range from 56 V to 66 V, at which the APD works in the linear mode. The measurement results are shown in Fig. 3. In the feasibility test, we choose two points beside the dashed line and record whether the APDs have a click event as shown in Fig. 4.
